# “Being with a Buddha”: A Case Report of Methoxetamine Use in a United States Veteran with PTSD

**DOI:** 10.1155/2017/2319094

**Published:** 2017-01-29

**Authors:** Joan M. Striebel, Emily E. Nelson, Raj K. Kalapatapu

**Affiliations:** ^1^Department of State Hospitals, Salinas Valley Psychiatric Program, 31625 Highway 101, P.O. Box 1080, Soledad, CA 93960, USA; ^2^Department of Psychiatry, University of California, 401 Parnassus Avenue, San Francisco, CA 94143, USA; ^3^San Francisco Veterans Affairs Medical Center, OTP Clinic, 4150 Clement Street, Building 1, Ground Floor, Room 24, San Francisco, CA 94121, USA

## Abstract

Methoxetamine (MXE) is a ketamine analogue with a high affinity for the N-methyl-D-aspartate (NMDA) receptor. MXE is a newly emerging designer drug of abuse and is widely available through on-line sources and is not detected by routine urine drug screens. In this report, we describe a United States (US) veteran with posttraumatic stress disorder (PTSD) and heavy polysubstance use, who injected high dose MXE for its calming effect. Given MXE's structural similarities to ketamine and recent work showing that ketamine reduces PTSD symptoms, we hypothesize that MXE alleviated this veteran's PTSD symptoms through action at the NMDA receptor and via influences on brain-derived neurotrophic factor (BDNF). To our knowledge, this is the first case report of self-reported use of MXE in the US veteran population. More awareness of designer drugs, such as MXE, is an important first step in engaging patients in the treatment of designer drug addiction in both military/veteran settings and civilian settings.

## 1. Introduction

We present a case of a young male United States (US) combat veteran with posttraumatic stress disorder (PTSD) and a complex substance use history, which included opioid use disorder treated with methadone, stimulant use disorder, and stimulant-induced psychosis. Initially unknown to clinical providers, he was also injecting large quantities of the designer drug methoxetamine (MXE) for its perceived calming effect. To our knowledge, this is the first case report of self-reported use of MXE in a US veteran [1].

## 2. Case Presentation

Mr. A is a 29-year-old US veteran with chronic PTSD (100% service-connected), heavy polysubstance use (opioid use disorder treated with methadone, stimulant [cocaine, methamphetamine] use disorder, sedative/hypnotic use disorder, hallucinogen use disorder (ketamine), cannabis use disorder, and tobacco use disorder), as well as unspecified depressive and anxiety disorders, childhood history of attention deficit/hyperactivity disorder, childhood sexual abuse, HIV on antiretroviral therapy, chronic hepatitis C, and tinnitus. He received care through the Veterans Affairs Medical Center (VAMC) and became a patient in the Opioid Treatment Program (OTP).

Three years after enrolling in the OTP, Mr. A began to describe symptoms of psychosis. These symptoms included hearing voices which talked about him, speaking his thoughts, and feeling as though someone was following him. His urine toxicology screen was positive for amphetamines, cocaine, cannabis, and opiates. Psychosis was treated with risperidone. Other medications included methadone 140 mg daily, emtricitabine 200 mg/tenofovir 300 mg daily, and raltegravir 800 mg daily. Over the ensuing six months, he was admitted to the VAMC psychiatric unit five times for treatment of acute psychosis. Medication trials for psychosis, depression, and anxiety included olanzapine, aripiprazole, mirtazapine, lorazepam, and gabapentin. Mr. A's methadone dose varied throughout this period depending on his concurrent substance use. At one point, he self-tapered methadone to discontinuation, although he later restarted it.

It was hypothesized that Mr. A's heavy use of stimulants precipitated the development of psychosis. This was based on the substances that he had reported using, the substances that were present on urine drug screens, and the observation that psychosis diminished rapidly during each hospitalization. Intermittent erythema and edema of his feet were noted, which were consistent with cellulitis and treated with antibiotics. Neurological examinations were normal during these episodes of cellulitis. Puncture marks were present on the dorsum of his left hand. There were no other abnormalities noted on physical examination.

Four years after enrolling in the OTP, Mr. A disclosed he was injecting MXE obtained from an on-line source. He described using MXE intermittently for the past year and had progressed to using 50–70 mg daily. He had tried ingesting it, but he preferred either intravenous or intranasal routes. When using MXE, he described feeling as if he was “being with a Buddha.” He preferred MXE to illicit use of ketamine, as he found MXE allowed him to feel more “spiritual” and “calm.” He believed that MXE relieved the distress and dysphoria of withdrawal that accompanied his methadone tapers, and he felt that using MXE allowed him to taper methadone more rapidly. The VAMC toxicology laboratory lacked the capability to detect MXE in urine. Thus, while urine drug screens returned positive for other substances, MXE was not identified.

Now five years after enrolling in the OTP, Mr. A continues to use MXE (70 mg, two to three times daily) along with other substances (methamphetamine, heroin, and alprazolam). Opioid replacement therapy was restarted, and his current dose of methadone is 50 mg daily. He is also prescribed olanzapine 15 mg twice daily to treat symptoms of psychosis.

## 3. Discussion

The discussion focuses on this veteran's PTSD and self-reported use of MXE to produce spiritual experiences and feelings of calm. This focus was chosen because, despite Mr. A's concurrent use of other substances with the potential to calm or produce spiritual effects, Mr. A attributed these effects to MXE. These effects were not reported prior to the addition of MXE to the plethora of substances he reported using. Mr. A's use of what he believed to be MXE was based on self-report. Novel psychoactive substances often contain compounds other than or in addition to those advertised [2, 3].

Mr. A is one of more than 1.8 million US troops who served in Operation Enduring Freedom (OEF) and/or Operation Iraqi Freedom (OIF) since combat began in 2001. Large-scale studies have found that 10–18% of OEF/OIF veterans are likely to have PTSD following deployment [4]. Substance use disorders (SUD) are extremely common in Iraq and Afghanistan veterans with PTSD and are associated with more severe PTSD symptoms and poorer outcomes across various domains [5].

**Figure 1:**
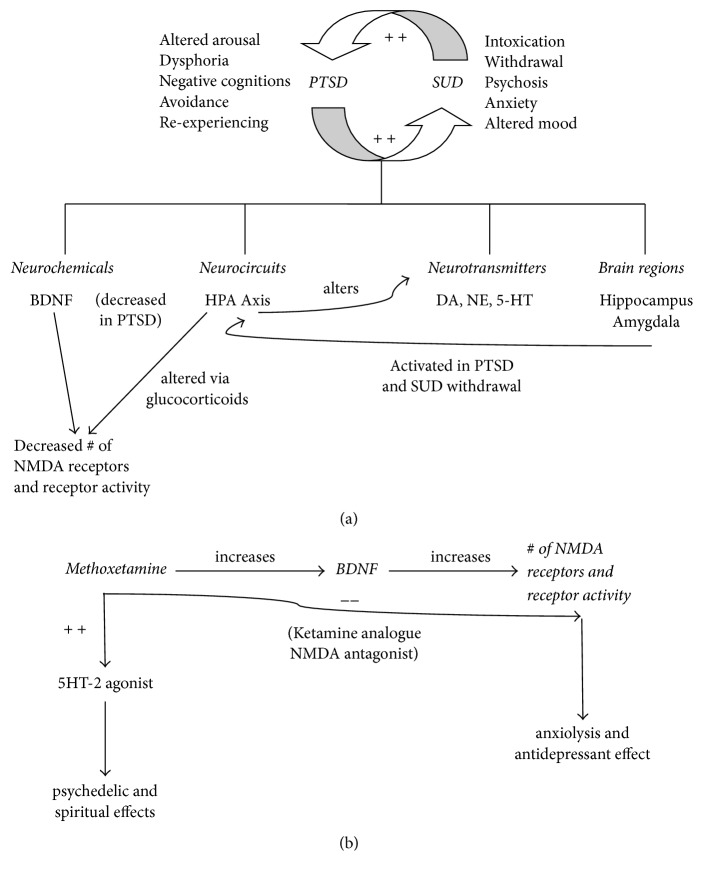
(a) Hypothesized Interplay of PTSD and SUD. (b) Downstream Effects of MXE.

Commonalities exist between neurotransmitters, brain regions, and neurocircuitry involved in PTSD and comorbid SUD (Figure 1(a)). Several key neurotransmitters, such as dopamine (DA), norepinephrine (NE), and serotonin (5-HT), play roles in reward, impulsivity, arousal, and anxiety [6]. Both PTSD and SUDs involve a learning and memory component, and the hippocampus and amygdala are key brain regions involved in both illnesses. Stress-induced activation of the hypothalamic-pituitary-adrenal (HPA) axis is seen in both diseases with the resultant release of corticotropin releasing factor (CRF) and dynorphin, an endogenous opioid peptide (Figure 1(a)). The CRF system plays a role in maintaining the negative mood states and anxious behaviors which resemble components of PTSD and which drive SUDs [7].

Other neurochemicals interact with the HPA axis in both PTSD and SUDs (Figure 1(a)). Brain-derived neurotrophic factor (BDNF) is a key polypeptide growth factor involved in processes required for long-term learning, memory, and conditioned drug reward [8]. BDNF appears to enhance fear extinction, and BDNF levels have been found to be significantly lower in individuals with PTSD [9].

BDNF signaling impacts the NMDA receptor, an ionotropic receptor located throughout the brain and involved in learning, memory, and long-term potentiation [10]. BDNF increases the number and activity of NMDA receptors on the plasma membrane of hippocampal neurons [11]. Ketamine, a noncompetitive NMDA receptor antagonist, diminishes anxiety [12] and depression [13]. Feder and colleagues [14] demonstrated that ketamine infusion resulted in reduction of PTSD symptoms in patients with chronic PTSD.

MXE is a ketamine analogue [15]. MXE is also a NMDA antagonist [16] and a 5HT2 agonist [1]. NMDA antagonism has been associated with the antidepressant effects of ketamine [17]. MXE is used for recreational and psychedelic effects and has been marketed as a “bladder-friendly” version of ketamine [18]. User Web reports and case reports describe the effects of MXE to be similar to those achieved with ketamine, although effects are often stronger and longer lasting [19]. High doses can result in hallucinatory experiences that users refer to as the “M-hole.” In their study of MXE user experiences, Kjellgren and Jonsson [20] identified ten themes, including spiritual and transcendent experiences, redosing, and addiction.

Not only did Mr. A suffer from chronic PTSD, he also suffered from the perceptual and cognitive impact of psychosis and the psychological and physical effects of sustained, heavy polysubstance use. Searching for a way to quiet this profound internal chaos, we propose Mr. A sought the calming effects of MXE. These effects served multiple purposes: (1) reducing the fear and anxiety associated with PTSD and psychosis; (2) mitigating the anxiety associated with the activation of the sympathetic nervous system due to opioid withdrawal; and (3) mitigating the anxiety and dysphoria due to the chronic activation of the HPA axis.

Given that MXE is a ketamine analogue, we suggest the anxiolytic and antidepressant effects resulted in part from an increased translation of BDNF, which in turn increased the expression of NMDA receptors and NMDA receptor activity. Blockade of these receptors by MXE resulted in anxiolysis and reduction of depression and PTSD symptoms (Figure 1(b)), analogous to the rapid effect of ketamine in mitigating depressive and PTSD symptoms. Mr. A's opioid use disorder was treated with methadone (an NMDA antagonist), which may have acted in concert with MXE to augment its calming effect. The 5HT2 receptor has been implicated in hallucinogen-induced feelings of spirituality and mysticism [21–23], and the spiritual feelings described by this patient may be due in part to MXE action at the 5HT2 receptor (Figure 1(b)).

In summary, this is the first case report of self-reported use of MXE in a US veteran. This veteran found MXE to exert calming effects and to allow spiritual experiences, which we hypothesize were due in part to increases in BNDF, its effect on the NMDA receptor, and MXE-induced antagonism of the NMDA receptor. Regarding military/veteran populations, a previous report commented that MXE may “threaten military readiness” [1]. This case report is of interest to US military/veteran medical providers, as well as providers working in civilian settings.
